# The Silent Valley Farm project in Kerala, India: a best-practice dairy farming system for the humid tropics

**DOI:** 10.1093/af/vfag017

**Published:** 2026-04-28

**Authors:** S Senthil Murugan, Graeme B Martin, Suraj Pullolil Thankappan, Prasad Ambazamkandi, Pramod Sathiamoorthy, Lasna Sahib, Manoj Madhusoodanan, Akhila Thampi C.

**Affiliations:** Livestock Research Station, Kerala Veterinary and Animal Sciences University, Thiruvazhamkunnu, Kerala 678601, India; UWA Institute of Agriculture and UWA School of Agriculture and Environment, The University of Western Australia, Crawley, 6009, Australia; Livestock Research Station, Kerala Veterinary and Animal Sciences University, Thiruvazhamkunnu, Kerala 678601, India; Livestock Research Station, Kerala Veterinary and Animal Sciences University, Thiruvazhamkunnu, Kerala 678601, India; Livestock Research Station, Kerala Veterinary and Animal Sciences University, Thiruvazhamkunnu, Kerala 678601, India; Livestock Research Station, Kerala Veterinary and Animal Sciences University, Thiruvazhamkunnu, Kerala 678601, India; Livestock Research Station, Kerala Veterinary and Animal Sciences University, Thiruvazhamkunnu, Kerala 678601, India; Livestock Research Station, Kerala Veterinary and Animal Sciences University, Thiruvazhamkunnu, Kerala 678601, India

**Keywords:** animal welfare, Attapady Black goat, heat stress management, tropical livestock systems, Vechur cattle

## Abstract

**Graphical Abstract:**
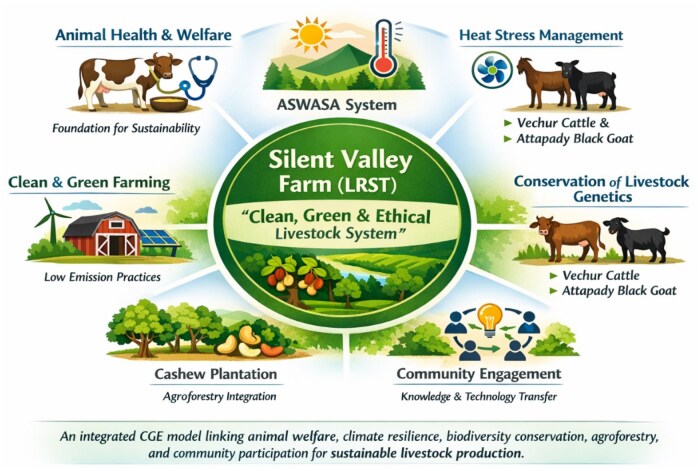

Implications

- The challenges for future farming are multi-disciplinary, and solutions are best developed and demonstrated on real-world farms that must produce food while also meeting the needs of the planet.
- The Silent Valley Farm addresses future livestock systems in an operational model of dairy production that simultaneously enhances animal productivity, health, and welfare, especially the mitigation of heat stress.
- We integrate dairy cattle management, goat and buffalo production with crop, fodder and silvopastoral systems as we seek to manage bio-diversity and landscape, conserve and manage water, reduce environmental impacts, and strengthen the socio-economic resilience of the local community.

## Introduction

Livestock systems are undergoing rapid transformation in response to climate change, increasing competition for land and water, heightened societal expectations regarding animal welfare, and the urgent need to reduce greenhouse gas emissions while maintaining food security (reviews: Martin, 2024; Maree et al., 2025). Dairy production systems, in particular, are under scrutiny because of their contribution to enteric methane emissions and their dependence on natural resources. Within this context, there is a growing demand for commercially viable, science-led demonstration farms that translate research outputs into practical, scalable solutions.

This paper outlines the major components of the *Silent Valley Farm* project on “Thiruvazhamkunnu,” the Livestock Research Station of the Kerala Veterinary and Animal Sciences University (KVASU). It is a full-scale, commercial “best-practice” farm that aligns and integrates land resource allocation, heat stress management, animal welfare, manure management, enteric methane mitigation, the carbon footprint of milk production, carbon farming, conservation-oriented projects, water resource stewardship, and community engagement. This multidisciplinary project provides science-based evidence for sustainable livestock development under humid tropical conditions and emphasizes system-level performance and replicability, thereby supporting informed decision-making by researchers, policymakers, and industry stakeholders.

In 2012, the *Silent Valley Farm* joined the Global Farm Platform (GFP), a network connecting research farms worldwide in a joint effort to address the challenges related to sustainable livestock production. In 2023, faculty from the *Silent Valley Farm* also participated in the GFP workshop on small-holder dairy systems that was held at the International Livestock Research Institute (Kenya). Participation in the GFP enables harmonized data generation, cross-regional comparisons, and global relevance for research outcomes generated by future farm projects. As an early member of the GFP, the *Silent Valley Farm* entered an alliance with a cognate project, *UWA Future Farm 2050* in Western Australia, that is described in a companion paper (Martin and Murugan, 2026). For these two model farms, the basic questions are the same, but the solutions differ because of major differences in geographical and socio-economic context.

## Site Context and Systems Rationale

**Figure 1.**
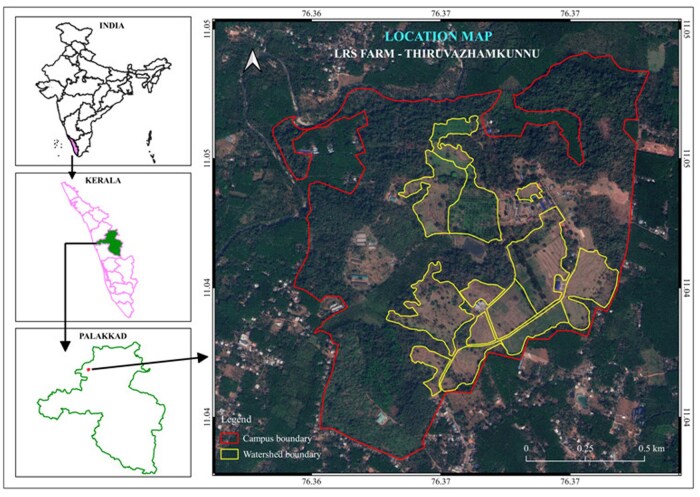
An aerial photograph of the KVASU Livestock Research Station (Thiruvazhamkunnu), the location of the *Silent Valley Farm* project. Red line: farm boundary. Yellow line: watershed boundary.

Thiruvazhamkunnu is a 163.3 ha farm nestled below the *Silent Valley* hills, a pristine evergreen forest region in the Western Ghats of southern India, in the Palakkad district of Kerala (11.04°N; 76.37°E), a humid, tropical agro-ecological zone (Figure 1). This zone is characterized by the typical Kerala rainfall pattern, dominated by the Southwest Monsoon (June–September) that brings rainfall ranging from 1376 mm (minimum during 2012) to 2583 mm (maximum during 2013), followed by a post-monsoon, dryer period (December–February) and lighter pre-monsoon showers (March–May). Rain is crucial for the water resources of the farm (wells, ponds, river), and the rainfall pattern drives a distinct seasonal sequence in the cultivation of fodder crops, and thus fodder quantity and quality. Thiruvazhamkunnu is therefore a site that represents climate-sensitive dairy production systems in the region.

## Land Resource Allocation and Whole-Farm Planning

Strategic land resource allocation underpins the sustainability of the project. It is guided by a systems-based approach that optimizes spatial allocation for livestock housing, fodder production, water-harvesting infrastructure, and ecological buffer zones. A major concern is wildlife encroachment, particularly elephants from the Silent Valley hills that damage cultivated fodder crops and create panic among laborers and local people. To address the problem, boundaries are demarcated between the farm and the adjoining local town, and KVASU installed a hanging electrical fencing system along 2 km of the boundary, cutting off a third of the total area, to reduce interactions between the elephants and the local people.

The soil type is mainly clay-kaolinitic with an acidic pH in the range of 4.9–5.4, electrical conductivity 0.080–0.089 dS/m, and organic carbon content 1.29–1.36%. Based on soil quality, water-holding capacity, and suitability for sprinkler irrigation, about 31% of the total arable area has been allocated to improved forage cultivation systems, including Bajra Napier hybrid (*Pennisetum americanum* x *Pennisetum purpureum*) cultivars (CO-3, CO-5) and giant Guinea grass, *Megathyrsus maximus* (Jacq.) (Simon and Jacobs, 2003). Plantation crops cover 26% of the land, mostly in silvo-pastoral systems incorporating arecanut (*Areca catechu* L.), coconut (*Cocos nucifera* L.), and cashew (*Anacardium occidentale* L.). About 28% of the land was identified as native forest and earmarked for initiatives aimed at enhancing conservation and biodiversity. Areas were also allocated for essential infrastructure (farm buildings, academic and residential facilities, laboratories, roads, storage units, training and research hostels, fencing, and water conveyance systems).

## Major Commercial Enterprise 1: “CGE” Dairy Production

The dairy industry is arguably the clearest demonstration of the value of ruminants for converting forage into high-quality food for humans. However, as with all livestock industries, we have seen the development worldwide of societal pressures (effectively market pressures) that have led to a vision for “clean, green and ethical” (CGE) livestock management: “clean” involves minimizing the use of drugs, chemicals, and hormones; “green” involves minimizing the impact on the environment (particularly, the greenhouse gas, methane); and “ethical” focuses on animal welfare. This concept was initially presented in 2004 in the context of small ruminant systems (updated by Martin, 2022), but the principles are applicable to other systems, such as dairy cattle (Martin et al., 2009).

The availability of diverse animal genetic resources, established infrastructure, and skilled technical personnel makes the *Silent Valley Farm* particularly suitable for long-term, systems-based research. There are dedicated facilities for conducting studies on animal welfare, reproductive performance, adaptation to heat stress, and management of health, enabling comprehensive evaluation of biological responses within sustainable farming systems. To this capability, we add geographical representation and global networking, as well as indigenous breed conservation (see below), and therefore strengthen the scientific and policy relevance of project outcomes across tropical livestock systems.

The major commercial enterprise on *Silent Valley Farm* is a herd of 250 Holstein–Friesian cross-bred dairy cattle. The characteristics of the genotype are maintained by using premium semen or sex-sorted semen supplied by Kerala Government institutions, following the breeding policy of the Government of India. “Farm fresh” milk is marketed to local people and to local co-operatives for processing and product sales.

### Animal health, nutrition, and welfare—a foundation for sustainability

Excellent animal health and welfare are directly linked to productivity, reproductive performance, longevity, and “methane efficiency” (see below). We are streamlining the systematic screening tests that are essential to ensure animal health, milk quality, reproductive efficiency, and biosecurity. Along with physical examination, lameness, dung, eye, nose, and body condition scores are checked at regular intervals (Mullan et al., 2020). Animals are screened for diseases like brucellosis and bovine tuberculosis at regular intervals and milk is routinely subjected to California mastitis tests, somatic cell counts, pH, and estimates of fat–solid-not-fat (SNF). These controls are critical for early detection of mastitis and to ensure compliance with food safety standards. Reproductive and metabolic screening, including using pregnancy diagnosis kits, progesterone profiling, and periodic evaluation of blood metabolites (calcium, phosphorus, ketone bodies), helps in early identification of infertility, milk fever, and ketosis. Regular parasitological screening through faecal egg counts and blood smear examination supports strategic deworming and vector control. In addition, water quality testing, analysis of feed and fodder for nutrient balance, along with the monitoring of environmental hygiene, form an integral part of herd-level management. Together, these processes provide a proactive approach for disease prevention, maximization of productivity and milk quality, and enterprise sustainability, and are therefore integral in a modern, welfare-oriented dairy farming system.

Effective bio-security is essential for sustaining productivity and reducing health-related risks. We have therefore integrated preventive bio-security measures such as controlled animal movement, hygiene protocols, routine health surveillance, and early disease detection. These measures are embedded within daily management practices, reducing disease incidence, minimizing antimicrobial use, and enhancing overall herd resilience.

### Heat stress

Under the prevailing tropical humid climate, there is a high risk that chronic heat stress will adversely affect animal health and productivity. At *Silent Valley Farm*, to mitigate thermal stress, priority is given to housing design (ventilation, allowance of adequate space) and continuous access to clean water. Nutrition, health management, and handling practices are also structured to support animal comfort and natural behavior.

The balance between heat generated by the animal and its ability to dissipate it is monitored with the widely used temperature-humidity index (THI). A trial at *Silent Valley Farm* during January to May 2023 (Singaravadivelan et al., 2025) showed that THI values frequently exceeded the discomfort threshold (>72), ranging from 75 in January to 82 in April, the period of peak heat stress, highlighting the dynamic environmental challenges faced by livestock in tropical climates. This analysis was supported by measurement of the temperature of the inner canthus and the respiratory rate in calves of Vechur (*Bos indicus*) and cross-bred (*Bos taurus × Bos indicus*) cattle, and Murrah buffalo (*Bubalus bubalis*). Inner canthus temperature was greatest in the cross-bred cattle, moderate in buffalo, and lowest in the Vechur cattle. The respiratory rate hierarchy was the same. These observations reflect fundamental differences in thermoregulatory strategies, with crossbred calves having a heightened susceptibility to heat stress, as they rely more on evaporative cooling mechanisms to dissipate heat (Singaravadivelan et al., 2025).

**Figure 2.**
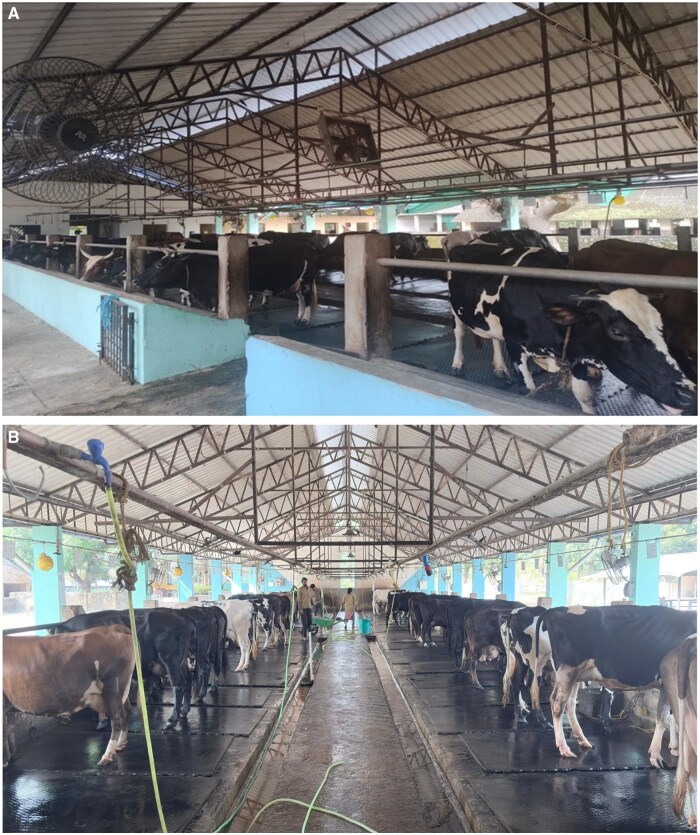
Implementation of the Automatic and Scientific Wetting for Animal Stress Alleviation (ASWASA) for Holstein–Friesian cross dairy cattle on the Silent Valley Farm.

To mitigate these impacts and enhance production efficiency, we implemented Automatic and Scientific Wetting for Animal Stress Alleviation (Figure 2). It is an automated system based on real-time monitoring of temperature, humidity, and the temperature humidity index (THI). When the THI exceeds the threshold of 78, the system first riggers rooftop sprinklers (1 per 5 metres of shed length) for 2–4 min, then activates foggers (1 per animal) for 30–45 sec, and finally operates fans for 15 min. The system repeats this cycle until the THI is below the threshold.

## Major Commercial Enterprise 2: Cropping (Cashew)

**Figure 3.**
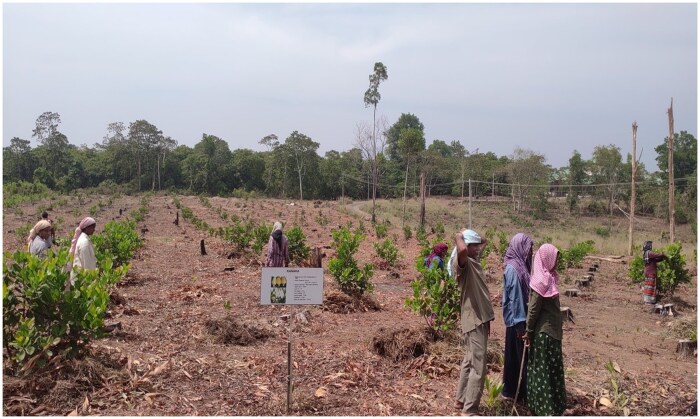
The high-density cashew plantation on *Silent Valley Farm* encourages diversification of farm income while promoting land use efficiency in an integrated crop-livestock silvipastoral system.

During 2023, in collaboration with the Directorate of Cashew Nut and Cocoa Development in the Ministry of Agriculture and Farmers’ Welfare (Government of India), 6–10 ha was developed as a frontline demonstration unit for high-density cashew plantation (Figure 3). The aim is to demonstrate the value of high-density planting, canopy regulation, and efficient soil and water management, while acting as a nucleus facility for the production and supply of scion wood and rootstock. The cashew plantation is integrated into the broader agroforestry–livestock system of *Silent Valley Farm* and promotes resource-efficient land use by demonstrating the value of providing shade to avoid evaporation of soil water, the use of recharge water and biomass residues, and under-storey forage. The outcome is enhanced system resilience, diversification of farm income, and strengthened linkages between perennial tree crops and sustainable livestock production.

## Conservation of Livestock Genetic Resources

**Figure 4.**
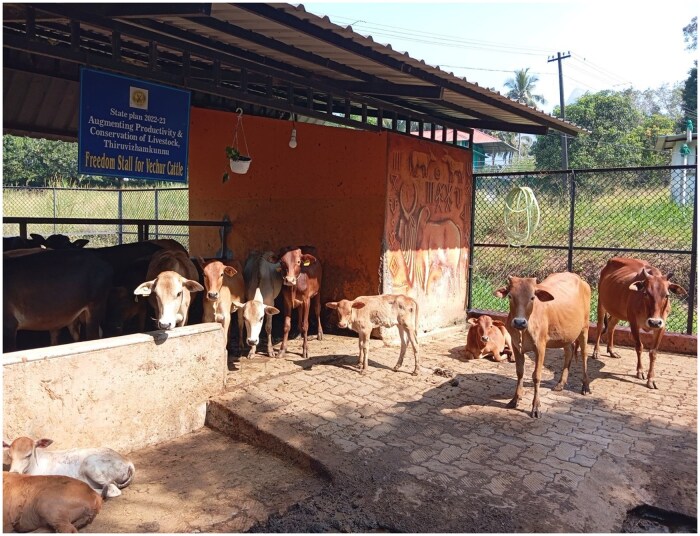
Vechur cattle at Silent Valley Farm.

**Figure 5.**
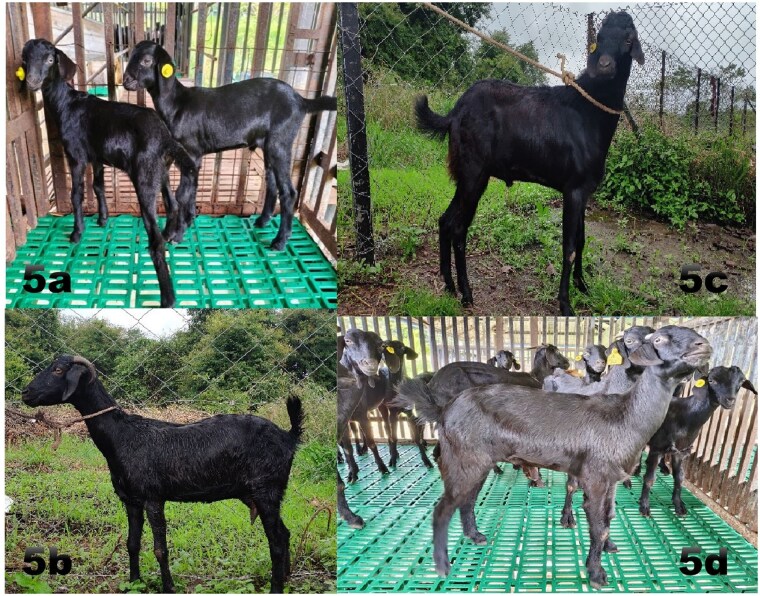
Attapady Black goats at *Silent Valley Farm*: (a) kids; (b) female; (c) male; (d) flock. They are kept on raised plastic floor platforms in a housing system, manually fed with 200–300 g of concentrates (pellets) and hybrid Napier grasses, and given water *ad libitum*.

According to FAO (2019), 26 % of the world’s indigenous animal breeds are in danger of extinction, increasing the risk that lack of genetic diversity will limit our efforts to combat climate change and disease, while raising productivity. The *Silent Valley Farm* project is therefore fulfilling obligations to conserve two genotypes that are local to Kerala—Vechur cattle (Figure 4) and Attappady Black goats (Figure 5). Both have high socio-cultural and ecological significance and their integration into the farm system allows the evaluation of productivity, resilience, methane emission intensity, and adaptive traits under best-practice management conditions.

The Vechur is a recognized Indian genotype from Kerala (Accession Number India cattle_0900_Vechur_03030; ICAR—National Bureau of Animal Genetic Resources). Its phenotypic characteristics are a tiny stature (average 107 kg), light red/black/gray coat color, high milk yield in proportion to body size, and production of A2 milk. The breed had almost become extinct by the 1980s due to a massive program of cross-breeding with exotic genotypes implemented by the government in order to enhance milk production. The Vechur cattle conservation program on *Silent Valley Farm* is funded by the Government of India through the Rashtriya Gokul Mission, and the population is now sustainable (Iype, 2013).

Importantly, with respect to the general aims of the Silent Valley Farm project, the changing climate underscores the importance of conserving locally adapted native breeds—we need to understand their inherent ability to thrive under challenging climatic conditions (Manoj et al., 2017). The Vechur genotype has already contributed by revealing genes for tolerance of temperature stress (Elayadeth-Meethal et al., 2021, 2023; Elayadeth-Meethal and Kolathingal-Thodika, 2025) and for resistance to infection (Kalaiyarasi and Elayadeth-Meethal, 2025). Non-genetic factors also play a crucial role in determining the reproductive and productive efficiency. Conception rates in Vechur cattle can vary from 32% in August to 61% in March. These estimates come from 868 records of breeding by natural service, spanning 2011–2024, in the conservation project at the Centre for Advanced Studies in Animal Genetics and Breeding in Mannuthy, Kerala (King et al., 2025).

The Attappady Black is a meat-type goat that is native to Kerala’s Attappady tribal hills. Due to crossbreeding and habitat loss, it has become critically endangered and the current population is less than 10,000. Through the Network Project on Animal Genetic Resources (AnGR), the Government of India supports its conservation, by supporting a population survey and by developing a Geographical Indication (GI) status. The Attappady Black herd on *Silent Valley Farm* is included as the multiplier unit of the network project. The Attappady Black goat is also expected contribute genetic traits associated with tolerance to thermal stress because it is superior to, for example, the Malabari goat, another native breed of Kerala (Thomas et al., 2015; Lakshmi et al., 2025). This work complements our efforts to strengthen the climate-resilience of livestock systems through targeted conservation, breeding, and sustainable grazing strategies.

## Management of Environment and Biodiversity

### Stewardship of water resources

Even in humid tropical regions, the seasonality and variability of rainfall can lead to periods of water scarcity that present significant challenges for livestock systems. During 2017, a comprehensive master plan was developed to optimize land and water resources through an integrated approach based on analysis of the watershed (Figure 1). The existing grasslands were delineated into 14 watersheds, thus facilitating targeted interventions for conservation of soil and water. These included the construction of 4.8 km of new waterways, 18.7 km of new contour bunds, renovation of 3.3 km of existing contour bunds, establishment of 10 water conservation structures, renovation of 1.2 km of stream channels, and implementation of rainwater harvesting through 24 rain pits across the farm.

Under the guidance of the ground-water authority of the Government of Kerala, the *Silent Valley Farm* project developed rainwater harvesting (ponds, wells, check dams, rainwater pits) and uses that water in an efficient irrigation system for fodder crops, and in the animal houses and the dairy where water meters record the quantities used for milking and cleaning. Slurry water is used for fodder cultivation. This approach has allowed significant reduction in the water footprint of milk production while maintaining animal health and hygiene standards.

### Methane—emissions reduction and emissions intensity

To reflect the global priority of reducing the impact of ruminant livestock on the climate, consideration of methane production is embedded within routine herd management and aligned with measures of productivity and economic performance. Rather than focusing solely on reduction of emissions, there is an emphasis on lowering “emissions intensity” (kg methane emitted per unit of milk produced; review: Martin, 2024). As well as the focus on health and welfare outlined above, emissions intensity is targeted through improving forage quality and digestibility, and precision feeding to enhance feed efficiency. Finally, we aim to optimize manure management to reduce anaerobic losses.

### Carbon footprint and carbon farming

In the context of emissions reduction, KVASU signed a Memorandum of Understanding (MOU) with Environmental Defense India Foundation (EDIF), a subsidiary unit of Environmental Defense Fund (an NGO based in the United States). This MOU supports implementation of research on the *Silent Valley Farm* that targets sustainable milk production through heat stress mitigation, carbon farming, and carbon credits, in accordance with the policies and regulations of the Government of India. Thus, quantifying and reducing the carbon footprint of milk production is a defining feature of the *Silent Valley Farm* project. Life cycle-based assessment frameworks are applied to account for emissions from feed production, enteric fermentation, manure management, and on-farm energy use.

In parallel, the project promotes carbon farming practices, including promotion of intensive cultivation of the perennial legume crop, hedge lucerne (*Desmanthus virgatus* (L.)), soil carbon sequestration through improved fodder management, integration of trees and perennial vegetation, and regenerative agriculture (Patil et al., 2025). Soil is sampled periodically at various depths from geo-referenced locations to evaluate the impact of sustainable fodder cultivation, manure recycling, and improved pasture management and baseline soil data on temperature, pH, and macro and micro minerals (calcium, phosphorus, potassium, magnesium, iron, manganese, copper, zinc, lead, molybdenum, boron, sodium, chromium, cobalt, cadmium). This framework also incorporates watershed interventions such as contour bunding, farm ponds, and rainwater harvesting to enhance soil moisture and carbon sequestration. Internal technical reviews ensure accuracy and transparency.

Manure management has increasingly been recognized as a critical component of sustainable dairy farming. At *Silent Valley Farm*, 25–40% of dung is placed into anaerobic digesters and the biogas produced is used to supply energy for the student canteen. Around 45% of dung produced is used in fodder plots, thus greatly reducing dependence on chemical fertilizers, which in turn reduce the dependency of chemical fertilizer to the extent of 60–65% and 1–5% of cow dung produced is dried during summer and sold to nearby farmers. These strategies enhance on-farm carbon sinks and contribute to balancing emissions, thereby supporting pathways toward low-emission or climate-neutral dairy systems. Efficient handling, storage, and utilization of manure minimizes nutrient losses, reduces emissions of methane and nitrous oxide, and protects soil and water quality while simultaneously enhancing nutrient recycling. Soil monitoring, described above, should provide an evidence base for future changes fodder cultivation practices.

Carbon farming is thus transforming the dairy system into a climate-positive production unit that contributes simultaneously to productivity, environmental sustainability, and climate change mitigation.

### Biodiversity

The *Silent Valley Farm* is considered a biodiversity-rich ecosystem. It supports collaborative agro-forestry projects, including the “All India Coordinated Research Project,” (Government of India). Bird survey studies conducted at *Silent Valley Farm* in 2026 recorded a total of 156 species, such as the Malabar Grey Hornbill (*Ocyceros griseus*), Heart Spotted Woodpecker (*Hemicircus canente*), and Indian Pitta (*Pitta brachyura*). The Indian giant squirrel (*Ratufa indica*), wild boar (*Sus scrofa*), bonnet macaque (*Macaca radiata*), and Indian Peafowl (*Pavo cristatus*) are commonly sighted. Documentation of these species justifies the allocation of a quarter of the farm for enhancing conservation and biodiversity.

## Community Engagement and Knowledge Exchange

Consistent with the broader agenda of social and economic sustainability, the *Silent Valley Farm* project emphasizes strong community relationships and knowledge exchange, by offering a demonstration and learning platform for farmers, students, extension professionals, and policymakers. Participatory engagement, transparency in performance outcomes, and capacity-building activities facilitate technology adoption and strengthen the trust between science and practice. This social dimension enhances the legitimacy and long-term impact of the model farm. On the *Silent Valley Farm*, most of the laborers engaged are from a 5 km radius of Thiruvazhamkunnu, and “farm fresh” milk is available for the local people to use in their households, and to shop owners and for mini restaurants/shops. The “farm fresh” milk is also sold to farm producer organizations and companies that process it and then market it in bottles in the peri-urban/urban area, thus improving their livelihoods. Thiruvazhamkunnu local traders also actively participate in auction sales of coconut, arecanut, and cashew nuts.

### Academic and research activities

A major research resource is the herds of crossbred dairy cattle, Murrah buffaloes, and Attapady Black and Malabari cross-breed goats, for which there is a well-integrated recording system of production and reproduction traits. The livestock are supported by a feed production system: fodders from cereal by-products (maize, sorghum), and 50 ha cultivated with several high-yielding fodder varieties, including cultivars of Bajra Napier hybrid (CO-3, CO-4) and giant Guinea grass, Para grass (*Brachiaria mutica* (Forsk) Stapf.), Congo-signal grass (*Brachiaria ruziziensis* Germain and Everard), Signal grass (*Brachiaria decumbens* Stapf.). and fodder trees, including Gliricidia (*Gliricidia sepium* (Jacq.) Walp.) and Subabul (*Leucaena leucocephala* (Lam.)). For ensiling grass, there are two bunker silos (80 t each) and seven pit silos (50 t each), supported by machinery (hay bailer, tractors, blower-type chaff cutter, feed mixing unit).

In the area of heat stress management for maximizing production and reproduction, 21 research projects were completed during the period 2021–2025 by bachelor, master, and doctoral students from the Veterinary Faculty, and B. Tech (Dairy) and Dairy Diploma students of our own and other universities. The students are also encouraged to enter internship training programs that increase their understanding of the best-practice systems management implemented on *Silent Valley Farm*.

### Extension facilities

There is a training facility with a hostel and a lecture hall with audio visual aids, where we offer hands-on practical training for farmers on dairy production, buffalo production, goat production, fodder production, fodder preservation, and training of labor skilled in animal husbandry.

## Conclusions

KVASU actively participates in research that contributes to a variety of critical global issues by connecting with three international research networks: the GFP, the World-wide Universities Network (WUN), and the Centre for One Health Education, Advocacy, Research and Training (COHEART). These networks allow us to focus on interactions among climate change, food security, food safety, zoonotic diseases, and sustainable farming. The *Silent Valley Farm*, as a member of the Global Farm Platform, is valued as a full-scale model farm that brings discussion of best-practice dairy farming for the humid tropics. It offers opportunities for evaluation and demonstration of innovative practices that integrate the major livestock and cropping enterprises while considering the management of natural resources, carbon emissions, the environment and biodiversity, and the conservation of rare native genotypes. Like all real farms, it engages with and supports its local community. There is real scope for expanding the experience and expertise of *Silent Valley Farm* to other geographical and socio-economic environments and thus greatly improving the options for “feeding the world without destroying the planet.”

## Author Biographies

**S. Senthil Murugan** earned his M.V.Sc. from Tamil Nadu Veterinary and Animal Sciences University, Tamil Nadu, and a PhD from Gandhi Gram Rural Institute (GRI) (Deemed University), India. He began his professional career as an Animal Nutritionist in the cattle feed industry and worked in various units of the Kerala Veterinary and Animal Sciences University (KVASU). He is currently serving as Unit Head and has used his experience to implement best-practice dairy farming systems at *The Silent Valley Farm*, Livestock Research Station, Thiruvazhamkunnu, KVASU. His primary research focus includes enhancing the quality of lingo-cellulosic fibrous materials to mitigate enteric methane emissions and developing area-specific mineral mixture to promote sustainable livestock production. His collaborative research work with the Board of Research in Nuclear Sciences (BRNS), Bhabha Atomic Research Centre (BARC), Mumbai, Government of India, and evaluation of fibrolytic enzymes through *in vitro* digestibility studies has been widely adopted in total ration formulations by dairy farmers. He has also developed encapsulated nutrients (probiotics, nano minerals, amino acids) as feed additives for ruminants at the lab level. He has been honored with a Gold Medal and recognition for the ICAR Best Teacher Award.

**Graeme B. Martin** gained a PhD from The University of Western Australia (UWA) and then worked in France (INRAE) and in Scotland (MRC) before returning to UWA. He subsequently reached the level of Professor (Chair) and is now Professor Emeritus. His research focused on how environmental factors affect reproduction, mostly in the sheep and the emu, using techniques in neuroscience, endocrinology, metabolism, behavior, reproductive technology, molecular biology, and computer modeling. He used his experience to develop “clean, green and ethical” livestock management, a theme he incorporated into “UWA Future Farm 2050.” He is a Lifetime Member of the *Society for Reproductive Biology* (Australasia) and a Fellow of the *Australian Association of Animal Sciences*. In 2019, he was elected as Associate Member of the *Academie d’Agriculture de France* and, in 2021, was awarded the 2021 Marshall Medal by the *Society for Reproduction and Fertility* (UK).

**P.T. Suraj** secured a PhD from the Tamil Nadu Veterinary and Animal Sciences University, India. He started his career at Farm Science Centre under Kerala Agricultural University (KAU) utilizing the great opportunity to work with the farmers for solving their problems. He later moved on to research farms and Veterinary College under Kerala Veterinary and Animal Sciences University (KVASU) and is presently working as Professor (Livestock Production Management) and coordinating research projects under the Silent Valley Farm Platform. His research areas include sustainable smallholder production, integrated farming systems, fodder production, and environmental stress management in cattle and goats.

**Ambazamkandi Prasad** has 25 years of professional experience including 8 years as veterinary surgeon in Animal Husbandry department of Kerala. He was a Co-PI and visiting scientist at University of Western Australia, Perth, Australia, and is recipient of Crawford Scholarship of Australia. He represented the LRST as a member of the Global Farm Platform in International Livestock Research Institute, Nairobi, Kenya. He is the course director of PG Diploma in Applied Animal Welfare, Ethics and Law and PG Diploma in Dairy Entrepreneurship and is coordinating the technology enabled distance learning center of the university. His specializations include livestock production management, animal welfare, and agricultural extension. He has received at the national level the Cherala Bhagya Raja Ram Award for Innovative Research in livestock production and Management and Kadambini Devi Award on Animal Behaviour of Indian Society of Animal Production and Management.

**S. Pramod** held positions like Teaching Assistant at Kerala Agricultural University and Animal Husbandry consultant at State Poverty Eradication Mission (Kudumbashree), Kerala. He was also employed as a Veterinary Surgeon in the State Animal Husbandry Department, with clinical and non-clinical roles like Technical Assistant to the District Animal Husbandry Officer. He obtained PhD from Kerala Veterinary and Animal Sciences University in Animal Genetics and Breeding with DST-INSPIRE fellowship of Government of India. He is working at Kerala Veterinary and Animal Sciences University as an Assistant Professor. His research interests include domestic animal diversity, breeding for disease resistance, thermal stress in ruminants, ruminant nutrition, and agronomy.

**Sahib Lasna** obtained her doctoral degree in Animal Nutrition from Kerala Veterinary and Animal Sciences University (KVASU), India, after completing her undergraduate training at Kerala Agricultural University and her postgraduate studies at Tamil Nadu Veterinary and Animal Sciences University. She is currently working as an Assistant Professor at the Livestock Research Station, Thiruvazhamkunnu, KVASU. Her doctoral research has focused on improving nutrient utilization and productivity in crossbred dairy cows under tropical conditions, with particular emphasis on straw-based total mixed rations. As part of this work, she developed a CNCPS-based feed library for 16 commonly used feed ingredients in Kerala, contributing to region-specific precision feeding strategies. Her broader research interests include ruminant nutrition, feed evaluation systems, and *in vitro* rumen fermentation methodologies. She has authored 15 peer-reviewed publications in reputed national and international journals.

**M. Manoj** earned his BVSc & AH from Kerala Agricultural University and completed his MVSc and PhD in Animal Genetics and Breeding from the National Dairy Research Institute (NDRI), Karnal, where he was awarded the Director’s Gold Medal for PhD and several merit recognitions. With over a decade of experience in teaching, research, and field-based livestock development, Dr. Manoj specializes in dairy cattle and buffalo breeding, conservation of animal genetic resources (especially Vechur and Kasargod cattle), frozen semen technology, and pig breeding. He has successfully handled several national- and state-funded projects, including those under the Rashtriya Gokul Mission and ICAR. He has guided PhD and postgraduate students, published extensively in reputed journals, and serves on editorial boards of veterinary science journals. His work significantly contributes to indigenous livestock conservation and genetic improvement in India.

**Thampi C. Akhila** obtained her PhD in Agronomy from Kerala Agricultural University, Thrissur, Kerala, India. Following brief academic appointments as Assistant Professor (Agronomy) at the Regional Agricultural Research Station, Pilicode, Kasaragod, and the College of Agriculture, Vellanikkara, Thrissur, under Kerala Agricultural University, she joined the Livestock Research Station, Thiruvazhamkunnu, Kerala Veterinary and Animal Sciences University, where she currently serves as Assistant Professor (Agronomy). Her research focuses on sustainable fodder production systems, with emphasis on hybrid Napier, crop intensification, stress tolerance, nutrient management, carbon sequestration, and climate change mitigation. She integrates field experimentation with soil–plant nutrient diagnostics and systems-based approaches to enhance productivity, resource-use efficiency, and resilience of forage-based livestock systems. She is a Life Member of the Indian Society of Weed Science (ISWS) and serves as a Co-Investigator of the Silent Valley Global Farm Platform Project, contributing to the development of climate-smart, resource-efficient, and sustainable livestock production systems.
